# The Charitable Dispensaries of Bengal

**Published:** 1902-01-25

**Authors:** 


### THE CHARITABLE DISPENSARIES OF BENGAL.

Although the charitable institutions of Bengal numbered in all 513 at the end of the year 1899, there were still many centres and places in the province which had not yet been provided with dispensaries. The establishment of such institutions no doubt involves a question of expenditure, but it is a matter of such extreme importance that it behoves both the local bodies and the people themselves to make every effort to increase the present total. The local bodies in most cases are fairly alive to the necessity of providing medical aid for the sick poor, but the Inspector-General of Civil Hospitals is very anxious that the people also should be made to understand that they must themselves second any efforts. It is difficult to state with any degree of accuracy the number of hospitals and dispensaries which are required. This must vary with the density of population, facilities of communication, amount of disease in the district, and other circumstances; but it is certain that, as a rule, no person should be more than fifteen to twenty miles from a dispensary, and that every town, village, or rural area, with a population of 3,000 persons or more, should have a dispensary with a properly qualified medical officer and the necessary establishment. The additions during 1899 to the existing total of charitable institutions was twenty-nine, of which eighteen were established by District Boards. These District Boards and Municipalities have usually great difficulty in meeting all the demands made upon them, but if more of the wealthy land-owners, especially those who do not reside on their estates, would contribute money for medical aid much more could be done in this direction. There are of course many whose generosity is well known, and who themselves maintain dispensaries where the medical aid is not confined to that given to their own servants, but relief is also afforded to all necessitous sick persons. The contributions of professional men, rich shopkeepers, and other leading inhabitants of the towns and large villages of Bengal are, however, far from liberal, and it is comparatively rare for them to render the assistance which they could so easily give, such as erecting a cottage hospital, endowing one either already existing, or paying the expenses of a bed. Further steps towards the prevention of disease are being tried by entrusting simple medicine chests to the head men of villages remote from dispensaries, and arranging for the visit of a medical officer to certain centres at short fixed intervals. These visits will be on market days, so as to extend the benefit to a greater number of persons, and extra visits will be made to fairs and places of pilgrimage, or wherever large assemblies of people congregate. With regard to the attendance at the hospitals during the twelve months ending in December, 1899, there was an increase of 184,000 in the number of Mohammedans, and 235,000 in the number of Hindus. The females, who were treated as indoor or outdoor patients, showed an increase of 55,000, and the children likewise attended in much larger numbers. The total number of operations performed during 1899 was about 14,500, an increase of 8,000—the deaths being 16 per cent. of the whole. The operations included extraction of lens, litholapaxies, lithotomies, and ovariotomies, in each of which classes there has been considerable improvement in numbers. This is, it is believed, partly due to satisfaction arising from the good results achieved from successful cataract cases, and in order that there may be no falling back, and also that a larger number of sufferers may benefit, the authorities are of opinion that it would sometimes be wise to pay the travelling expenses of poor men and women who would be willing to be operated on for cataract, if the cost of travelling did not stand in the way. Care will, of course, have to be taken that the assistance is only given to those who really need it, as the Lieutenant-Governor thinks it most undesirable that anything should be done which might have the appearance of offering inducements to people to accept relief of this kind. Moreover, there are comparatively few villages which are so far removed from a dispensary as to make such a course really desirable.

